# Exposures to 2,4-Dichlorophenoxyacetic acid with or without endotoxin upregulate small cell lung cancer pathway

**DOI:** 10.1186/s12995-021-00304-4

**Published:** 2021-04-17

**Authors:** Geetika Kaur, B. V. Sunil Kumar, Baljit Singh, R. S. Sethi

**Affiliations:** 1 Department of Animal Biotechnology, College of Animal Biotechnology, Guru Angad Dev Veterinary and Animal Sciences University, Ludhiana, Punjab 141004 India; 2 Department of Microbial and Environmental Biotechnology, College of Animal Biotechnology, Guru Angad Dev Veterinary and Animal Sciences University, Ludhiana, Punjab 141004 India; 3 Western College of Veterinary Medicine, University of Saskatchewan, Saskatoon, S7N 5B4 Canada

**Keywords:** 2,4-D, SCLC, p53, LPS, Lungs, Apaf1

## Abstract

**Background:**

Pesticide residues in food and environment along with airborne contaminants such as endotoxins pose health risk. Although herbicide 2,4-Dichlorophenoxyacetic acid (2,4-D) has been associated with increased risk of lung cancers such as small cell lung cancer (SCLC) among agricultural workers, there are no data on the SCLC signaling pathway upon 2,4-D exposure without LPS or in combination with endotoxin.

**Methods:**

We exposed Swiss albino mice (*N* = 48) orally to high (9.58 mg kg^− 1^) and low (5.12 mg kg^− 1^) dosages of 2,4-D dissolved in corn oil for 90 days followed by *E. coli* lipopolysaccharide (LPS) or normal saline solution (80 μl/animal). Lung samples and broncho-alveolar fluid (BALF) were subjected to Total histological score (THS) and total leucocyte count (TLC) and differential leucocytes count (DLC) analyses, respectively. We used microarray and bioinformatics tools for transcriptomic analyses and differentially expressed genes were analyzed to predict the top canonical pathways followed by validation of selected genes by qRT-PCR and immunohistochemistry.

**Results:**

Total histological score (THS) along with BALF analyses showed lung inflammation following long term dietary exposure to high or low doses of 2,4-D individually or in combination with LPS. Microarray analysis revealed exposure to high dose of 2,4-D without or with LPS upregulated 2178 and 2142 and downregulated 1965 and 1719 genes, respectively (*p* < 0.05; minimum cut off 1.5 log fold change). The low dose without or with LPS upregulated 2133 and 2054 and downregulated 1838 and 1625 genes, respectively. Bioinformatics analysis showed SCLC as topmost dysregulated pathway along with differential expression of Itgb1, NF-κB1, p53, Cdk6 and Apaf1. Immunohistological and quantitative real time PCR (qRT-PCR) analyses also supported the transcriptomic data.

**Conclusions:**

Taken together, the data show exposures to high and low dose of 2,4-D with/without LPS induced lung inflammation and altered pulmonary transcriptome profile with the involvement of the SCLC pathway. The data from the study provide the insights of the potential damage on lungs caused by 2,4-D and help to better understand the mechanism of this complex relation.

**Supplementary Information:**

The online version contains supplementary material available at 10.1186/s12995-021-00304-4.

## Introduction

Agricultural workers are commonly exposed to pesticides during production, transportation, planning and utilization of pesticides [1]. Among pesticides, herbicides are used to kill weeds and are frequently used (36%) followed by insecticides (25%), fungicides (10%) and other pesticides (29%) [2]. Since the registration of 2,4-D for use as herbicide in 1940s, its use has grown globally for controlling broad-leaved weeds in a variety of settings such as crops and other vegetation on rangelands, lawns, golf courses, roadways, parks and forests to aquatic settings [3]. It mimics the growth hormone auxin (Indole acetic acid) resulting in uncontrolled and unorganized growth and eventually death of weeds [4]. It commonly persists in water bodies as it does not easily biodegrade in aquatic environments [5]. Therefore, the frequent use of 2,4-D leads to environmental contamination and exposure to the non-target species in many ways such as inhalation, ingestion, and dermal routes [6].

Exposure to 2,4-D can cause damage to non-target species as it interferes with chemical reactions, enzymes activity, cell functions and DNA structure causing mutation, free radical formation and apoptosis [4]. The damage includes increased risk of non-Hodgkin’s lymphoma [7], Parkinson [8] and various cancers including soft-tissue sarcoma, bladder and respiratory cancers among frequently exposed farmers and factory workers [9, 10]. Exposures to 2,4-D increased the expression of oxidative stress markers such as 8-hydroxy-2′-deoxyguanosine (8-OHdG) and 8-isoprostaglandin-F_2α_ (8-isoPGF) among Iowa corn farmers suggesting that 2,4-D may play important role in the pathogenesis of chronic diseases including cancer among agricultural workers [11–13].

Endotoxins are ubiquitously present in our environment including that in the agricultural settings [14, 15]. Agricultural workers may also potentially get exposed to both pesticides and environmental bacterial lipopolysaccharides (LPS). Previous studies from our laboratory indicate that endotoxin interaction with various classes of pesticides increases the pesticide induced lung damage [16–20]. LPS ligate Toll-like Receptor 4 (TLR-4) to activate various signaling pathways including NF-κB leading to the production of inflammatory mediators and lung inflammation [21]. LPS may also promote tumor growth in vivo by amplifying the release of proinflammatory cytokines [22]. Currently, there is scarcity of data on the activation of lung cancer pathways following chronic exposure to 2,4-D without LPS or when co-exposed with endotoxin/LPS.

Lung cancer is the main cause of cancer-related death around the world and small cell lung cancer (SCLC) accounts between 13 and 15% of diagnosed lung cancers resulting in about 250,000 death worldwide yearly [23]. SCLC arises from neuroendocrine cells in the bronchial epithelium and is characterized by rapid tumour growth, high vascularity, genomic instability and early metastatic dissemination [24]. The development of SCLC disrupts normal DNA repair mechanisms by involving signature genes including p53, Itgb1, Cdk6, NF-κB1 and Apaf1. These genes alone or in collaboration with others influence various behaviours such as activation, invasiveness, cell cycle and cell death of tumor cells [25–32]. Currently, there are no data on the expression of SCLC signature genes in animals exposed to 2,4-D without LPS or in combination with LPS.

Further, there is very little known of expression of genes involved in cancer and inflammation following exposure to 2,4-D without LPS or combined with LPS. Therefore, we tested the hypothesis that 2,4-D induces lung inflammation and up-regulates SCLC pathway by using a microarray approach along with bioinformatics tool followed by protein analyses of selected genes. We report the first data that 2,4-D exposure dysregulates SCLC pathway with increased protein expression of Itgb1, NF-κB, p53 and Cdk6 and decreased expression of Apaf1.

## Material and methods

Chemicals Used: Technical 2,4-Dichlorophenoxyacetic acid, Plant Culture Tested (Catalogue no. PCT0825) with purity > 95% was obtained from Himedia, Nashik. Corn oil (Catalogue no. C8267) and lipopolysaccharide (LPS) from *Escherichia coli* (CAS no L3129) were obtained from Sigma-Aldrich, Bengaluru, India. Primary antibodies anti mouse p53, Itgb1, Cdk6, Nfkb1, Apaf1 (Elabsciences) and secondary antibody horseradish peroxidase-conjugated was purchased from Santa cruz. The others chemicals included Trizol reagent (Life Technologies), c-DNA first strand synthesis kit (Thermo Scientific, USA) and colour development commercial kit (SK4100; Vector Laboratories).

### In vivo experiments

Institutional Animal Ethics Committee (IAEC), Guru Angad Dev Veterinary and Animal Sciences University (GADVASU), Ludhiana approved the experimental protocols with guidelines from Committee for the Purpose of Control and Supervision of Experiments on Animals (CPCSEA). Total forty-eight (*N* = 48) Swiss albino healthy male mice of age 6–8 weeks and the average weight between 28 and 35 g were procured from Disease-Free Small Animal House, Lala Lajpat Rai University of Veterinary and Animal Sciences, Hisar, Haryana, India. Three mice in each polypropylene cage were maintained with 12 h light and 12 h dark cycle at small animal housing hall, GADVASU, Ludhiana. The animals were given synthetic pelleted diet in the morning and evening and water ad libitum. The feed contained black grams, soya based rich in nutrients, vitamins and other essential parameters. These mice were acclimatized for 1 week prior to the start of the experiment.

### Experimental design

Animals (*N* = 48) were randomly divided into two treatments (high and low) and one control group (*N* = 16 in each group). The biomonitoring data on urine samples provide the most reliable information on human exposure to 2,4-D [33, 34] and the available data suggest that 9.02 mg/l is the mean level of in 2,4-D the urine samples of exposed farmers corresponding to 0.2 mg/kg/d exposure [35, 36] Based on previous data on dietary exposures in rat indicating 5 mg kg^− 1^ as no observable effect level (NOEL) for chronic exposures [37], we selected high (9.58 mg kg^− 1^ body weight of mouse per day) and low (5.12 mg kg^− 1^ body weight of mouse per day) dose of 2,4-D. The treatment groups were administered high and low dose of 2,4-D by gavage needle orally dissolved in corn oil for 90 days. Corn oil was orally administered by gavage needle to control group. At the end of experiment eight animals from each group were anaesthetized with xylazine ketamine combination anaesthesia (1/10th of the body weight) and challenged with LPS@ 80 μl/animal intranasally. The remaining mice were administered 80 μl of normal saline solution (NSS) intranasally. We use LPS 80 μg/animal, the amount is sufficient to elicit the lung injury as reported earlier [16, 20]. Animals were sacrificed with xylazine ketamine combination (0.1 μl/10 g of body weight) after 9 h of LPS/NSS challenge. Hence, the study included six different groups viz. control, LPS only, high dose without LPS, low dose without LPS, high dose with LPS and low dose with LPS.

### Body weight analysis

The change in body weight was calculated as described previously [20]. Briefly the initial body weight (at the start of the experiment) was subtracted from the final body weight (at day 90 of experiment) and expressed as average body weight gain.

### Collection of samples

The blood sample was collected by cardiac puncture and bronchioalveolar lavage (BAL) fluid was collected from left lung [17]. BAL fluid was centrifuged at 500 g for 10 min at 4 °C to collect pellet and supernatant. The supernatant was stored at − 80 °C for further analysis and the pellet resuspended in PBS for total leukocyte count (TLC) and differential leukocyte count (DLC) analysis on the same day. Right lung samples were stored in RNA later solution at − 80 °C for RNA isolation. Left lung was fixed in situ in paraformaldehyde solution and used for histopathology and immunohistochemistry.

### Total leukocytes and differential leukocyte count analysis

Blood and BAL fluid samples were processed for TLC and DLC analysis on the same day as described earlier [16]. Briefly, 380 μl of the white blood cell diluting fluid was mixed with 20 μl of the blood/BAL fluid for TLC analysis. For DLC analysis clear blood smear was prepared and stained with Leishman stain. Neutrophils and lymphocytes were counted on each slide at 40X. About 100 cells per sample were identified and counted by an evaluator blinded to the identity of the samples and count was expressed as absolute number of neutrophils and lymphocytes per microlitre of blood.

### Hematoxylin and eosin staining

The paraformaldehyde fixed lung (6 animals from each group) was processed to obtain 5 μm thick paraffin sections which were stained with hematoxylin and eosin for histopathological analysis. Pathological features (peribronchial infiltration, perivascular infiltration, blood vessel congestion, increase in perivascular space, thickening of alveolar lining and inter alveolar oedema) were graded to obtain cumulative total histology score (THS) in a blinded fashion as described previously [38] Further, each feature was scored from 0 to 3 based on its absence (0) or presence to a mild (1), moderate (2) and severe (3) degree.

### Microarray gene expression and analysis

About 50 mg of right lung from each animal was used to isolate RNA using the Trizol method (Ambion, Life Technologies, USA). Two RNA samples from each group were randomly used for microarray analysis. The purity and concentration of extracted RNA was checked by using the Nanodrop spectrophotometer (Thermo Fisher). The quality check of the total isolated RNA was also performed in Agilent 2100 Bioanalyzer using the Agilent RNA 6000 Nano Kit. RNA samples with an RNA Integrity Number (RIN) > 7 were selected for microarray analysis. Microarray analysis was performed using the mouse microarray slide of format 8 × 60 K (ID No: 0307760384; Agilent Technologies). Labelling of total RNA (100 ng) was done with Low Input Quick Amp WT Labelling Kit followed by hybridization and scanning. After generating the microarray scan images, the feature extraction was done by Feature Extraction software version 10.7.3. Data generated were further analyzed by Genespring version 14.9 to identify the differentially expressed genes (DEGs) with cut off of 1.5 log fold change and *p* < 0.05.

### Functional annotation and kyotoencyclopedia of genes and genomes (KEGG) pathway enrichment analysis

Gene Ontology (GO) enrichment analysis including molecular function, biological processes and cellular components was performed on DEGs as well as uniquely expressed genes by the Database for Annotation, Visualization and Integrated Discovery (DAVID), a web-based bioinformatics tool (https://david.ncifcrf.gov/) by using *Mus musculus* as background and default options and annotation settings. Further gene lists containing gene identifiers (probe set IDs) and corresponding expression values (fold change) were uploaded to DAVID Bioinformatics Resources (version 6.7) to identify top dysregulated pathways.

### Quantitative real-time PCR (qRT-PCR)

Microarray data for the mRNA expression of p53, Itgb1, Cdk6, NF-κB1 and Apaf1 was validated by qRT-PCR. Briefly, 400 ng/μl of total RNA from six animals from each group was reversed transcribed into cDNA using a Revert transcriptase cDNA synthesis kit (Thermo Scientific). qRT-PCR was performed using SYBR green chemistry with published primer sequences for p53 gene [39], Itgb1 [40], NF-κB1 [41], Apaf1 [42] and β- actin as an endogenous control [42]. Primer sequences for Cdk6 was self-designed. The relative expression of each sample was calculated by using the ΔΔCT methods [43].

### Immunohistochemistry

The paraffin lung sections were subjected to immunohistochemical staining as described earlier [44, 45]. The sections were stained with primary antibodies (rabbit polyclonal) against mouse p53 (E-AB-32468; dilution 1:20), Itgb1 (E-AB-10403; dilution1:50), Cdk6 (E-AB-10222; dilution 1:10), NF-κB1 (E-AB-35022; dilution 1:25) and Apaf1 (E-AB-15478; dilution 1:10) followed by appropriate horseradish peroxidase conjugated secondary antibody (Polyclonal goat anti-rabbit; Santa cruz; dilution 1:400). The colour development was carried out with commercial kit (SK4600, Vector Laboratories, USA) and methyl green was used as a counter stain. Controls included staining without primary antibody.

### Grading for immunohistochemistry

Immuno-positive p53, Itgb1, Cdk6, NF-κB1 and Apaf1 cells were counted in the lung tissue sections of five animals from each group. The cells were counted manually in 10 fields/section in an area of 0.2mm^2^ under the 40X objective lens of the microscope so as to maintain the uniformity [44, 45]. The evaluator was blinded to the identity of treatment groups.

### ELISA (enzyme-linked Immunosorbent assay)

Flat-bottomed (Nunc, Maxisorp) plate was coated with BAL fluid diluted in coating buffer (Carbonate-bicarbonate buffer, pH 9.3) and incubated at 4 °C, overnight. BAL fluid was discarded followed by addition of 100 μl blocking buffer (2.5% Skimmed milk powder in PBS) and incubation at 37 °C for 1 h. Plates were washed thrice with PBS-T (pH 7.4). Primary antibody (50 μl) (p53, Itgb1, Cdk6, NF-κB1 and Apaf1; dilution 1:20 in blocking buffer) was added into each well and incubated for 1 h at 37 °C. After incubation the plate was washed thrice with PBS-T buffer (pH 7.4) and incubated with 50 μl horseradish peroxidase-conjugated secondary antibody (dilution 1:100 in blocking buffer) at 37 °C for 1 h. Following thrice washing with PBS-T (pH 7.4), 200 μl of OPD substrate (dissolved in phosphate –citrate buffer, pH 5.0) was added into the wells and kept at room temperature till the color appeared (2–3 min). The reaction was stopped by adding 50 μl of 3 M H_2_SO_4_ and the absorbances were recorded at 490 nm in a Synergy Hi Hybrid Reader (Bio Tek).

### Statistical analysis

Data from TLC, DLC, histopathology, immunohistochemistry, ΔCT values and ELISA were presented as mean ± Standard Error (SE). Data were subjected to two-way analysis of variance (ANOVA) followed by Tukey’s post-hoc test, using GraphPad Prism software (evaluation version). We considered a *P*-value of < 0.05 to significant.

## Results

### Average body weight gain

There was increase in the average body weight in all the groups after 90 days compared to initial body weight without any significant changes among all groups and no mortality was observed throughout the experiment.

### Total leukocyte count and differential leukocyte count analysis

*Blood:* Exposure to LPS or high (9.58 mg kg^− 1^) dose of 2,4-D without LPS increased TLC of blood along with increase in neutrophils count and decrease in lymphocytes count. Further, exposure to high dose of 2,4-D combined with LPS increased TLC compared to LPS alone (Table 1). Although treatment with low (5.12 mg kg^− 1^) dose of 2,4-D without LPS did not alter the TLC of blood, but in combination with LPS increased TLC compared to control and individual low dose group. There was increase (*p* < 0.05) in neutrophils count and decrease in lymphocytes count following exposure to low dose of 2,4-D without LPS or in combination with LPS compared to control group.

**Table 1:** Total leukocyte count (TLC) and Differential Leukocyte count (DLC) of blood and BAL fluid (per μl) following exposure to 2,4-D with or without endotoxin

Experimental Groups		Blood		BAL Fluid	
	TLC (per μl)	Absolute number of Neutrophils (%)	Lymphocytes (%)	TLC (per μl)	Absolute number of Neutrophils (%)
Control	3699.16 ± 199.1^a^	28 ± 1.06^a^	72 ± 1.06^a^	157.83 ± 3.49^a^	21.83 ± 1.92^a^
LPS	4951.66 ± 219.7^b^	39.5 ± 1.05^b^	60.5 ± 1.05^b^	437 ± 15.52^b^	35.33 ± 1.72^b^
High dose of 2,4-D	4748.83 ± 289.8^b^	36.16 ± 1.83^b^	63.83 ± 1.83^b^	329.83 ± 28.72^c^	30.16 ± 1.16^c^
Low dose of 2,4-D	4483.33 ± 162.6^a^	34.66 ± 2.18^b^	65.33 ± 2.18^b^	303.83 ± 12.1^c^	30.5 ± 1.64^c^
High dose of 2,4-D + LPS	5826.66 ± 160.5^c^	51.83 ± 0.98^b^	48.16 ± 0.98^b^	492.16 ± 15.61^b^	42.16 ± 1.66^b^
Low dose of 2,4-D + LPS	5100.83 ± 136.9^b^	45.66 ± 3.52^b^	54.33 ± 3.52^b^	432.33 ± 17.8^b^	38.83 ± 2.18^b^

TLC and DLC expressed as Mean ± SE

^a,b,c^no common superscript between two levels of an effect indicates significant difference (p < 0.05)

6 animals from each group were used

### Bronchoalveolar lavage fluid

LPS increased (*p* < 0.05) the TLC and neutrophils in BAL fluid compared to control group. Similarly, high or low dose of 2,4-D increased (*p* < 0.05) the TLC compared to control and LPS group and neutrophils compared to control (Table 1). Further, high or low dose when combined with LPS increased (*p* < 0.05) TLC and neutrophil count compared to individual high or low group, respectively.

### Histopathological examinations

**Fig. 1:**
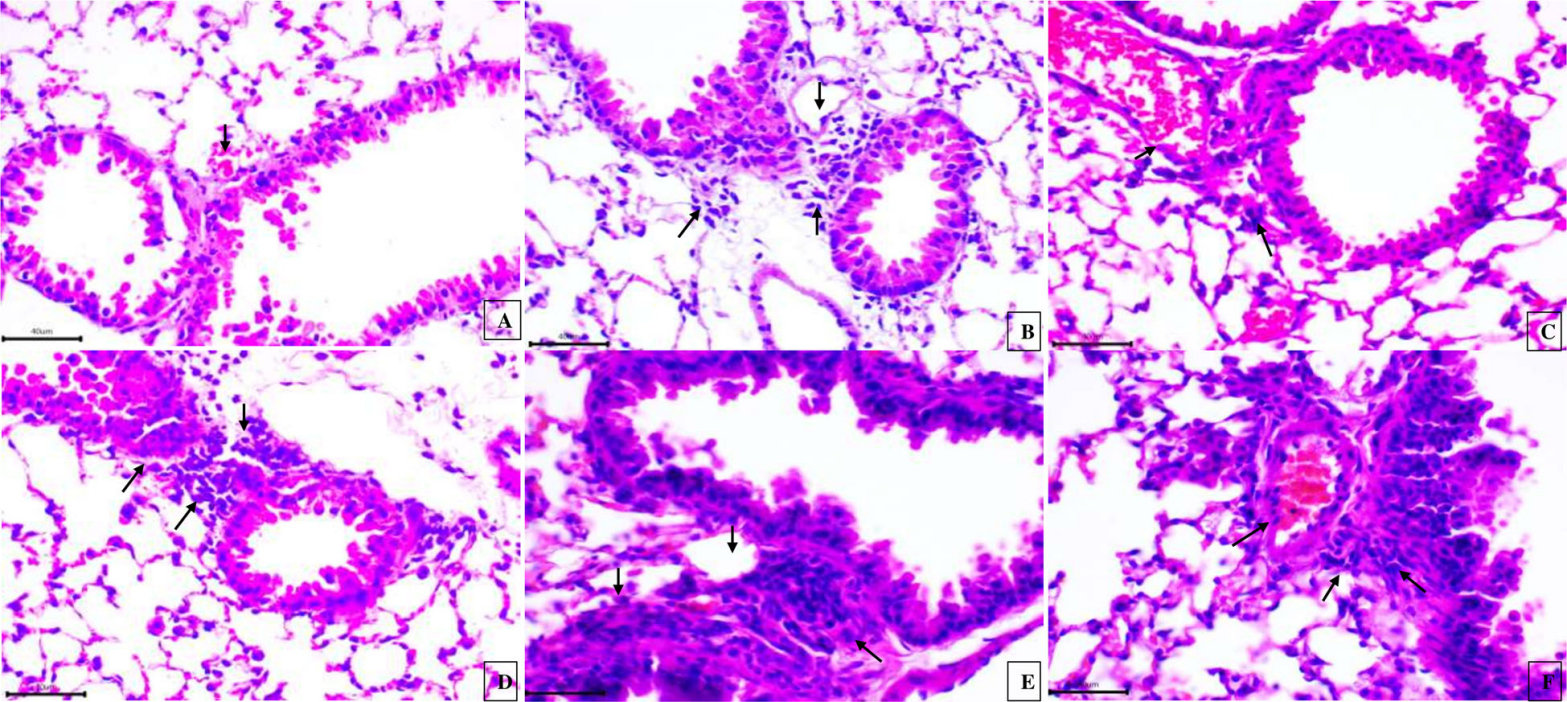
H&E staining: Paraffin section of lung showing normal alveolar epithelium (double arrows) and alveolar septa (single arrow) in control group (**a**). Perivascular (single arrow) and peribronchial (double arrows) infiltration of mononuclear cells following exposure of LPS (**b**), high dose of 2,4-D without LPS (**c**), low dose of 2,4-D without LPS (**d**), high dose in combination with LPS (**e**), low dose in combination with LPS (**f**). Haematoxylin and Eosin staining. Original magnification: 40X; 6 animals from each group were used

Hematoxylin and eosin stained lung sections from the control mice showed normal histoarchitecture (Fig. 1a). Exposure to LPS, high or low doses of 2,4-D individually or combined with LPS treatments caused lung inflammation characterized by congestion in blood vessels, peribronchial and perivascular accumulation of mononuclear cells and increase in the total histological score (THS) in all the treatment groups compared to the control (Fig. 1b-f; Suppl Table 1).

### Differentially expressed genes (DEGs) and functional analysis

**Fig. 2:**
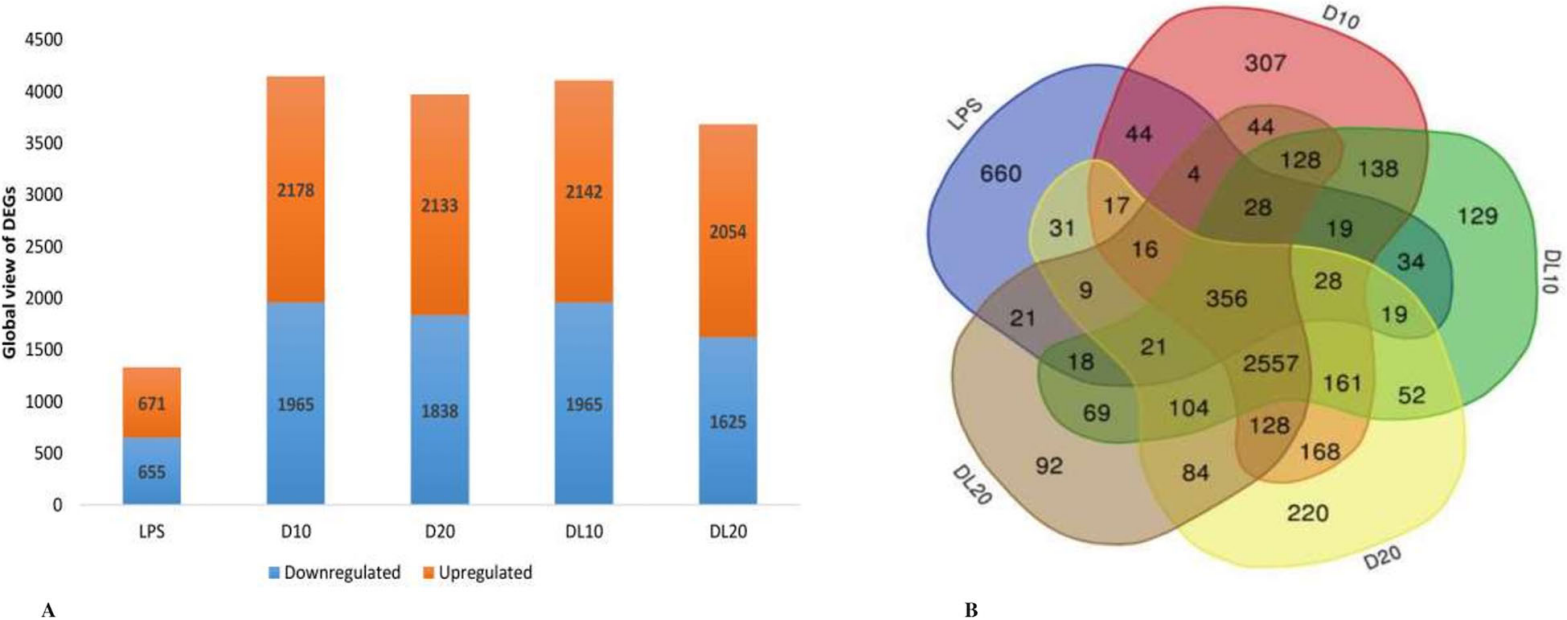
Hierarchical clustering and overlap of DEGs: Global view of Differentially Expressed Genes (DEGs) in different groups (**a**), Venn diagram (**b**) showing the overlap of DEG’s involved across LPS, high dose of 2,4-D without LPS (D10) or in combination with LPS (DL10), low dose of 2,4-D without LPS (D20) or in combination with LPS (DL20) group

A total of 5351 genes were differentially expressed (*p* < 0.05; fold change > ±1.5) following exposure to LPS and high or low dose of 2,4-D without LPS or a combination of 2,4-D and LPS. LPS treatment alone upregulated 671 genes and down-regulated 655 genes. Treatment with high dose (9.58 mg kg^1^) and low dose (5.12 mg kg^− 1^) of 2,4-D caused the upregulation of 2178 and 2133 genes and downregulation of 1965 and 1838 genes, respectively. Further 2,4-D in high and low dose in combination with LPS up regulated 2142 and 2054 genes and down regulated 1719 and 1652 genes, respectively as compared to control group (Fig. 2a). The gene overlap studies of differentially expressed genes (DEG) in all the groups showed 356 (216 upregulated and 140 down-regulated) commonly expressed genes in all the treatment groups compared to control. The relative expression levels of these genes are illustrated as a Venn diagram (Fig. 2b).

### Biological classification and pathway enrichment analysis of DEGs

Gene ontology enrichment analysis revealed that DEGs were significantly enriched in genes involved in various biological processes including response to oxygen containing compound, tissue development, regulation of protein modification process, regulation of cell population proliferation and nucleic acid metabolic process (Suppl Table 2). KEGG pathway enrichment analysis revealed that SCLC pathway was the topmost dysregulated pathway following exposure to high or low dose of 2,4-D with or without LPS. KEGG pathway enrichment analysis also suggested that p53, Itgb1, Cdk6, NF-κB1 and Apaf1 genes were hub genes primarily associated with SCLC pathway.

### Validation of microarray data by qRT-PCR and immunohistochemistry

#### p53

**Fig. 3:**
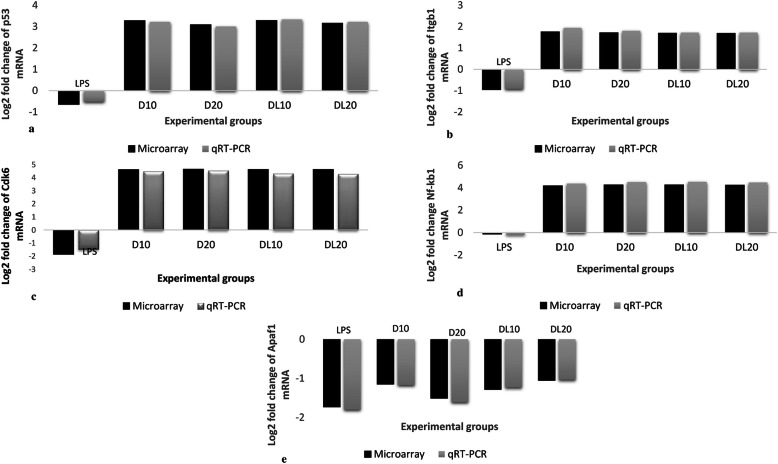
mRNA expression of qRT-PCR and microarray data: Fold change concordance of p53 (**a**), Itgb1 (**b**), Cdk6 (c), Nfkb1 (**d**) and Apaf1 (**e**) by qRT-PCR

Lung transcriptomic analysis revealed the up regulation of *p53* mRNA following exposure to high or low doses of 2,4-D with or without LPS. LPS did not alter the mRNA expression of p53. However, there was 3.28, 3.09, 3.29 and 3.16 folds increase in the mRNA expression of p53, respectively, following exposure to high dose, low dose, high dose in combination with LPS and low dose in combination with LPS. The qRT-PCR data were found to be in concordance with the microarray data (Fig. 3a).

**Fig. 4:**
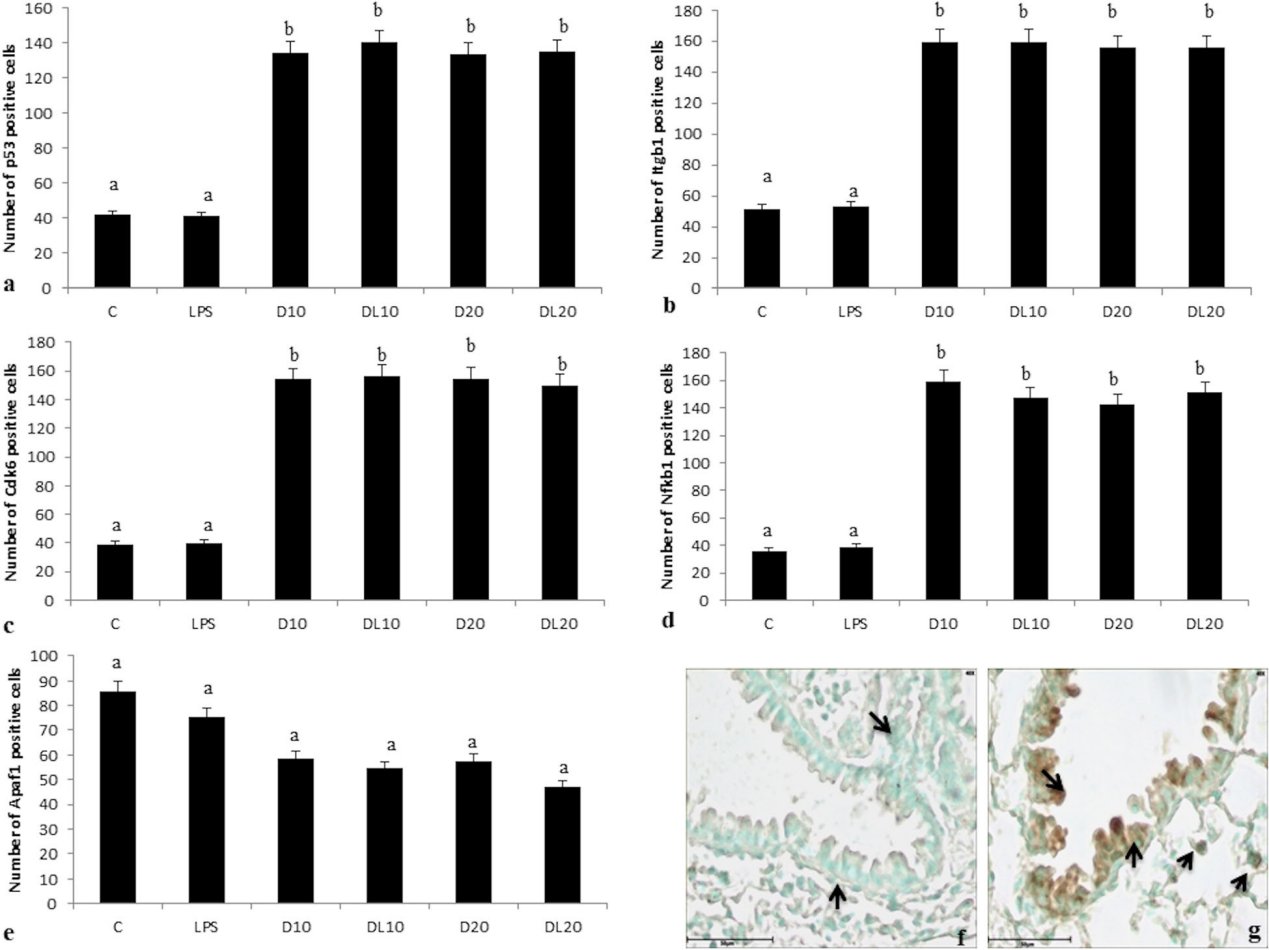
Quantification of immunopositive cells a) p53, b) Itgb1, c) Cdk6, d) Nfkb1 and e) Apaf1 in control (C), LPS, high dose without LPS (D10), low dose without LPS (D20), high dose in combination with LPS (DL10) and low dose in combination with LPS (DL20) group. Lung section stained without primary antibody (f) does not show any colour development in airways epithelium and staining with p53 (g) showed immunopositive reaction in airway epithelial (arrow) and septal cells (arrow head). ^a,b^ no common superscript between two levels of an effect indicates significant difference (*p* < 0.05)

The lung sections incubated without primary antibody did not show any colour development (Fig. 4). Lung tissues from control and LPS-treated mice showed weak staining for p53 in the airways epithelial and alveolar septal cells (Suppl Fig. 1). However, the high or low doses of 2,4-D without LPS or in combination with LPS showed strong reaction for p53 (Suppl Fig. 1). There was a significant increase in the number of p53-positive cells in lungs of mice exposed to both doses of 2,4-D compared to control and LPS group (Fig. 4a). Further, high or low dose in combination with LPS significantly increased the number of p53 cells compared to LPS group.

#### Integrin β1

Global view of DEG’s revealed down-regulation of integrin β1 (− 0.94 fold) by LPS alone and up regulation following exposure to high (1.76 fold) or low (1.71 fold) doses of 2,4-D without LPS. There was increase in integrin β1mRNA expression following exposure to, high dose (1.69 fold) and low dose (1.68 fold) in combination with LPS. The qRT-PCR data were found to be in concordance with the microarray data (Fig. 3a).

There was a mild staining for integrin β1 protein in the airway epithelial cells, alveolar septal cells and occasionally in large septal cells/macrophages in lungs from control and LPS group (Suppl Fig. 2). While LPS alone didn’t change the expression of the integrin, the high or low doses of 2,4-D without LPS or in combination with LPS caused an increase in intensity and number of cells positive for integrin β1 (Fig. 4b and Suppl Fig. 2).

#### Cdk6

Lung transcriptomic analysis revealed the up regulation of *Cdk6* mRNA following exposure to high or low doses of 2,4-D with or without LPS. Treatment with LPS downregulated (− 1.85 folds) Cdk6 mRNA. However, there was increase in the mRNA expression of Cdk6 following exposure to high dose (4.61 folds), low dose (4.64 folds), high dose in combination with LPS (4.62 folds) and low dose in combination with LPS (4.62 folds). The qRT-PCR data were found to be in concordance with the microarray data (Fig. 3c).

Lung sections from the mice in control and LPS groups showed weak to mild reactivity for Cdk6 in the airways epithelial and alveolar septal cells (Suppl Fig. 3). However, the high or the low dose without LPS or in combination with LPS increased the number of lung cells positive for Cdk6 compared to the control and LPS group (Fig. 4c).

#### NF-κB1

Microarray analysis revealed the upregulation of NF-κB1 gene following exposure to high and low doses of 2,4-D with or without LPS. However, LPS reduced NF-κB1 mRNA expression by − 0.12 folds. There was increase in the expression NF-κB1 following exposure to high dose (4.16 folds), low dose (4.25 folds), high dose in combination with LPS (4.24 folds) and low dose in combination with LPS (4.23 folds). The qRT-PCR data were found to be in concordance with the microarray data (Fig. 3d).

A mild NF-κB1 staining was localized in the airway epithelial cells and alveolar septal cells in lungs of mice from control and LPS group (Suppl Fig. 4). The high or the low dose without LPS or combined with LPS induced strong staining in alveolar epithelium cells, alveolar septal cells and macrophages compared to the control and LPS group (Suppl Fig. 4). LPS exposure did not cause any change in the number of NF-κB1 positive lung cells compared to control group. There was a significant increase in the number of NF-κB1 positive cells in lungs of mice exposed to high or low dose of 2,4-D compared to control and LPS group (Fig. 4d). Further, high or low dose in combination with LPS significantly increased the number of immunopositive NF-κB1 cells compared to LPS group but did not vary from individual high or low treatment group, respectively (Fig. 4d).

#### Apaf1

Lung transcriptomic analysis revealed the down regulation of Apaf1 mRNA following exposure to high or low doses of 2,4-D with or without LPS. Treatment with LPS downregulated Apaf1 mRNA by − 1.73 folds. Further, the Apaf1 mRNA decreased in lungs of mice treated with high dose (− 1.15 fold), low dose (− 1.51 fold), high dose combined with LPS (− 1.28 fold) and low dose combined with LPS (− 1.05 fold). The qRT-PCR data were found to be in concordance with the microarray data (Fig. 3e).

Lungs from control mice showed strong staining for Apaf1 in the alveolar cells (Suppl Fig. 5). LPS exposure also showed moderate to strong Apaf1 reactivity in the alveolar cells (Suppl Fig. 5). Further lungs from the mice exposed to the high or the low dose without LPS or in combination with LPS showed moderate reactivity for Apaf1 protein in alveolar cells (Suppl Fig. 5). There was a significant decrease in the number of Apaf1 positive cells in lungs of mice exposed to high or low dose of 2,4-D compared to control and LPS group (Fig. 4e). Further, high or low dose in combination with LPS significantly decreased the number of immuno-positive Apaf1 cells compared to LPS group but did not vary from individual high or low treatment group, respectively (Fig. 4e).

### Expression of proteins in BAL fluid

**Fig. 5:**
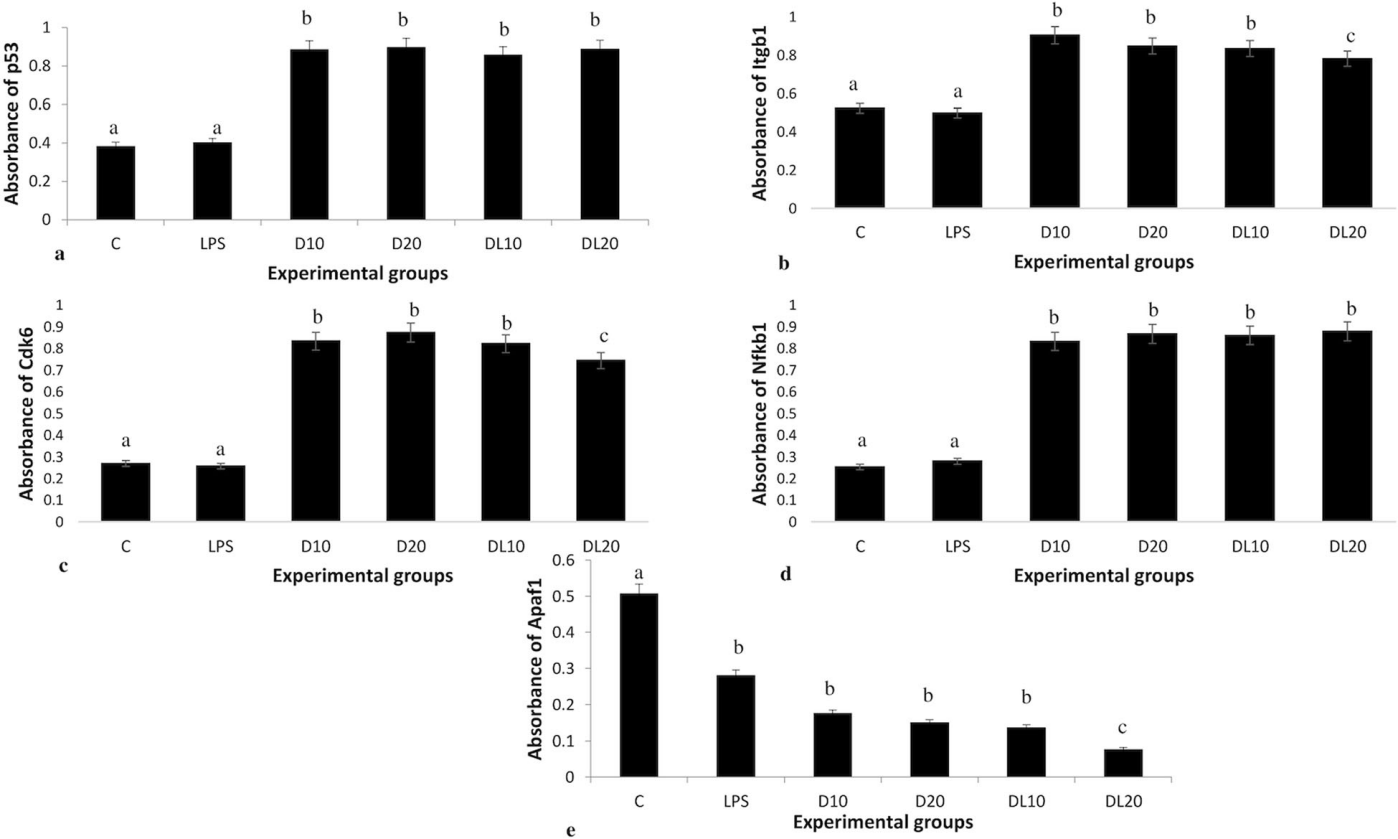
ELISA: Absorbances reflecting concentration of p53 (a), Itgb1 (b), Cdk6 (c), Nfkb1 (d) and Apaf1 (e) in BAL fluid in control, LPS, high dose without LPS (D10), low dose of 2,4-D without LPS (D20), high dose in combination with LPS (DL10) and low dose in combination with LPS (DL20) group. ^a,b,c^ no common superscript between two levels of an effect indicates significant difference (p < 0.05)

Indirect ELISA was carried out to compare the relative differences in absorbances as a readout of concentrations of p53, Itgb1, Cdk6, NF-κB1 and Apaf1 proteins in the BAL fluid (Fig. 5). LPS treatment did not alter the protein concentration of p53, Itgb1, Cdk6 and NF-κB1 in BAL fluid compared to control group. Exposure to the high or low dose of 2,4-D increased (*p* < 0.05) the BAL fluid concentration of p53 (0.886, 0.898 folds), Itgb1 (0.905, 0.848 folds), Cdk6 (0.833, 0.874 folds) and NF-κB1 (0.833, 0.867 folds) compared to the control and LPS groups, respectively. LPS decreased the concentration of Apaf1 compared to control. Further there was a decrease (*p* < 0.05) in the concentration of Apaf1 (0.176 and 0.150 folds) following exposure to the individual high or low dose as compared to control and LPS group. Furthermore, exposure to the low dose of 2,4-D in combination with LPS decreased (*p* < 0.05) the concentration of Apaf1 (0.077) as compared to individual low dose group (Fig. 5).

## Discussion

We present new data indicating lung inflammation and SCLC pathway as the top most dysregulated pathway along with altered expression of Itgb1, NF-κB1, p53, Cdk6 and Apaf1 following exposure to 2,4-D with or without LPS. These data add to understanding of potential role of 2,4-D in the development of lung cancer in the agricultural workers.

First of all, we wanted to understand whether exposure to 2,4-D without LPS or in combination with LPS causes lung inflammation. We used LPS exposure alone as a control and its exposure caused lung inflammation as reported previously [17, 20]. We used a combination of BAL analyses and Total Histological Score (THS) to determine lung inflammation. Because activated blood cells migrate into lung alveoli through coordinated actions of adhesion molecules and chemokines, BAL analysis is a reliable marker of lung inflammation [22]. High and low dose of 2,4-D with or without LPS caused significant increase in the TLC of BAL fluid. There was increased peribronchial and perivascular infiltration of mononuclear cell in the lung and significant increase in the THS in all groups compared to the control. Herbicide 2,4-D had been previously reported to cause lung injury [46]. Our previous work also showed long term dietary exposure to pesticide such as fipronil [17, 47], ethion [20], lindane [19], imidacloprid [16] and indoxacarb [48] cause lung injury. In addition, similar to observations made with other pesticides [16, 49, 50], there was increase in blood TLC, neutrophils and lymphocytosis with the high and low dose without LPS to indicate systemic immune response. The histopathological and BAL fluid data taken together show that both high and low doses of 2,4-D irrespective of co-exposure with LPS inflamed the lungs and combination with LPS did not show any synergistic effect.

The major focus of the work however was to explore the lung responses to 2,4-D exposure considering there is epidemiological linkage of lung cancer with exposure to 2,4-D [13]. There was differentiation in the effects of exposure of high dose of 2,4-D without LPS as it globally dysregulated higher number of genes compared to control group. Among the dysregulated genes, SCLC pathway was the topmost dysregulated pathway along with upregulation of p53, Itgb1, Cdk6, NF-κB1 and downregulation of Apaf1 following exposure to the high or the low dose of 2,4-D without LPS or in combination with LPS. We examined the expression of p53, Itgb1, Cdk6, NF-κB1 and downregulation of Apaf1 at the mRNA and protein levels in the lung to further understand their potential roles in 2,4-D induced cellular changes in the lung.

The p53, Itgb1, Cdk6, NF-κB1 and Apaf1play important roles in cell signaling and functions including cell cycle regulation and repair. High or low doses of 2,4-D without LPS upregulated the p53 and decreased Apaf1 mRNA and protein expression in the lung. However, combination of high or low dose with LPS did not alter the p53 and Apaf1 expression compared to individual high or low group without LPS. p53 trans-activates expression of genes involved in apoptosis, cell cycle regulation, and DNA damage repair [51]. The tumor suppressor gene, p53, plays critical role in preventing cancer development [52] and is the most frequently altered gene in around 50% of cancers in human such as breast, colon, lung, liver, prostate, bladder and skin cancer [25]. This tumor suppressor *p53* gene plays an important role in the onset of SCLC development by enabling the invasion, metastasis, proliferation and cell survival of malignant cells [53], Apaf1 is involved in apoptosome formation and its low expression is observed in aggressive tumors [54] and occurs in lung tumors such as NSCLC [55]. There is a close linkage between p53 and Apaf1 in chronic myeloid leukemia patient as APAF-1 is a transcriptional target of p53 [56]. Taken together, dysregulation of p53 and Apaf1 may underly in induction of lung cancer following exposure to 2,4-D.

Both high and the low doses of 2,4-D without LPS significantly increased Itgb1 expression in the lung tissues along with its increase in BAL fluid. However, addition of LPS to high or low dose did not change the mRNA expression of Itgb1 compared to individual high or low group without LPS. Interestingly, integrin β1 promotes cell adhesion to the extracellular matrix and is associated with highly invasive and metastatic behaviour in SCLC [27]. The increased expression of Itgb1 is correlated with poor prognosis in lung cancers [57]. Further there was increase in the expression of Itgb1 protein in macrophages, alveolar epithelium and alveolar septal cells. Other integrins such as αvβ3, a5β1 and αvβ6, are expressed at low or undetectable levels in adult epithelia, but their expression increases in some tumors [58]. The increased expression of Itgb1 may activate macrophages and facilitate their surveillance role in the lungs [59] and have a role in neutrophil recruitment [60]. The data suggest that high or low dose of 2,4-D without LPS caused a significant increase in the Itgb1 expression in lungs and BAL fluid indicating 2,4-D induced chronic inflammatory changes and may promote formation of lung tumor.

Integrins such as Itgb1 in addition to their roles in activation of inflammatory cells also phosphorylate and activate the focal adhesion kinase resulting phosphorylation of the cyclin D/Cdk4/6 complex via ERK pathway to promote the cell cycle progression [61]. Cdk6 is frequently amplified or overexpressed in a variety of human tumors [62]. We observed upregulation of Cdk6 protein expression in lungs following exposure to high or low dose of 2,4-D without LPS and LPS in combination high or low dose did not show any synergistic effect compared to individual high or low group without LPS. Aberrant expression of Cdk6 protein has been reported in many tumors suggesting that Cdk6 protein promotes tumor progression [63] and contributes to chronic inflammation and neoplasia through NF-κB [64]. The data indicate pulmonary upregulation of Cdk6 during 2,4-D induced lung damage which may induce lung tumorigenesis.

Inflammation and angiogenesis proceed in a coordinated manner and sustain one another during wound healing and tissue repair in many chronic inflammatory diseases and in cancer [22]. NF-κB pathways are important in broad range of cellular processes including inflammation and cancer progression [65]. NF-κB expression is elevated in Kras induced lung adenocarcinomas and blocking NF-κB significantly reduces tumor growth in mice [66]. Blockade of NF-κB activity is associated with suppression of angiogenesis, invasion and metastasis [60]. NF-κB1 is a pleiotropic transcription factor that promote tumor cell invasion and angiogenesis by regulating expression of various factors that very important in tumorigenesis including matrix metalloproteinases, cyclooxygenase-2 (COX-2), iNOS, chemokines and inflammatory cytokines [67]. Exposure to high and low doses of 2,4-D without LPS increased the mRNA expression of NF-κB1, increased concentration in BAL fluid and protein expression in the lung. High or low dose in combination with LPS did not alter the mRNA expression of NF-κB1 compared to individual high or low group without LPS. NF-κB expression increases during SCLCs as compared to NSCLCs [31] and in lung damage induced by hyperoxia [68], oxidative stress [69] and in number of pulmonary disease including cystic fibrosis, pulmonary hypertension and cancer [70]. The data taken together suggest that high or low dose of 2,4-D without LPS or in combination with LPS caused a significant increase in the NF-κB1 expression in lungs and BAL fluid.

Taken together, we provide the first evidence of activation of genes, Itgb1, Cdk6, NF-κB1, p53 and Apaf1, involved in SCLC signaling pathway and induction of lung inflammation in animals exposed to 2,4-D. These data are important because SCLC is the dominant cause of patient death [71] and 2,4-D is linked to pulmonary cancer. These descriptive data set the stage for mechanistic studies involving methods such as gene-knockout mice and in vitro methods such as siRNA.

## Conclusion

We conclude that long-term exposure to high (9.58 mg kg^− 1^) and low (5.12 mg kg^− 1^) dose of 2,4-D without LPS significantly alter the histoarchitecture. Further, we observed that LPS alone did not cause any significant alteration in transcriptome profiling, however, 2,4-D without LPS significantly alter the transcriptome profile of lungs compared to control and LPS groups. Moreover, bioinformatics analysis reveals the involvement of the SCLC pathway as the top-most dysregulated pathway and the data are significant because of altered expression of key genes associated with this pathway viz. Itgb1, Cdk6, NF-κB1, p53 and Apaf1 during chronic exposure of 2,4-D induced lung damage.

## Supplementary Information

**Additional file 1: Figure S1.** Immunohistochemistry for expression of p53: Immunopositive reactivity for p53 in alveolar septal cell (single arrow) and epithelium cells (double arrow) in control (A), LPS (B), high dose without LPS (C), low dose without LPS (D), high dose in combination with LPS (E) and low dose in combination with LPS (F) group. Original magnification: 40X.

**Additional file 2: Figure S2.** Immunohistochemistry for expression of Itgb1: Immunopositive reactivity for Itgb1 in alveolar septal cell (single arrow) and epithelium cells (double arrow) in control (A), LPS (B), high dose without LPS (C), low dose without LPS (D), high dose in combination with LPS (E) and low dose in combination with LPS (F) group.. Original magnification: 40X.

**Additional file 3: Figure S3.** Immunohistochemistry for expression of Cdk6: Immunopositive reactivity for Cdk6 in alveolar septal cell (single arrow) and epithelium cells (double arrow) in control (A), LPS (B), high dose without LPS (C), low dose without LPS (D), high dose in combination with LPS (E) and low dose in combination with LPS (F) group. Original magnification: 40X.

**Additional file 4: Figure S4.** Immunohistochemistry for expression of Nfkb1: Immunopositive reactivity for Nfkb1 in alveolar septal cell (single arrow) and epithelium cells (double arrow) in control (A), LPS (B), high dose without LPS (C), low dose without LPS (D), high dose in combination with LPS (E) and low dose in combination with LPS (F) group. Original magnification: 40X.

**Additional file 5: Figure S5.** Immunohistochemistry for expression of Apaf1: Immunopositive reactivity for Apaf1 in alveolar septal cell (single arrow) and epithelium cells (double arrow) in control (A), LPS (B), high dose without LPS (C), low dose without LPS (D), high dose in combination with LPS (E) and low dose in combination with LPS (F) group. Original magnification: 40X.

**Additional file 6: Table S1.**

**Additional file 7: Table S2.**

## Data Availability

The datasets during and/or analysed during the current study are available from the corresponding author on reasonable request.
