# Correction to: Exposures to 2,4-Dichlorophenoxyacetic acid with or without endotoxin upregulate small cell lung cancer pathway

**DOI:** 10.1186/s12995-021-00307-1

**Published:** 2021-05-04

**Authors:** Geetika Kaur, B. V. Sunil Kumar, Baljit Singh, R. S. Sethi

**Affiliations:** 1 Department of Animal Biotechnology, College of Animal Biotechnology, Guru Angad Dev Veterinary and Animal Sciences University, Ludhiana, Punjab 141004 India; 2 Department of Microbial and Environmental Biotechnology, College of Animal Biotechnology, Guru Angad Dev Veterinary and Animal Sciences University, Ludhiana, Punjab 141004 India; 3 Western College of Veterinary Medicine, University of Saskatchewan, Saskatoon, S7N 5B4 Canada

**Correction to: J Occup Med Toxicol 16, 14 (2019)**

**https://doi.org/10.1186/s12995-021-00304-4**

Following the publication of the original article [1], we were notified of a mistake in the indicated dose of 2,4-D:

- Incorrect: “high (9.58 mg kg-1) and low (5.12 mg kg-1)”
- Correct: “high (37mg kg-1) and low (18.5 mg kg-1)”

This correction does not affect any part of the results or the discussion in the manuscript.

The original article has been corrected.
