# Supplementary figures and images for: Exposures to 2,4-Dichlorophenoxyacetic acid with or without endotoxin upregulate small cell lung cancer pathway
Source: J Occup Med Toxicol. 2021 Apr 17;16:14. doi: 10.1186/s12995-021-00304-4 (PMC8052721; doi:10.1186/s12995-021-00304-4)

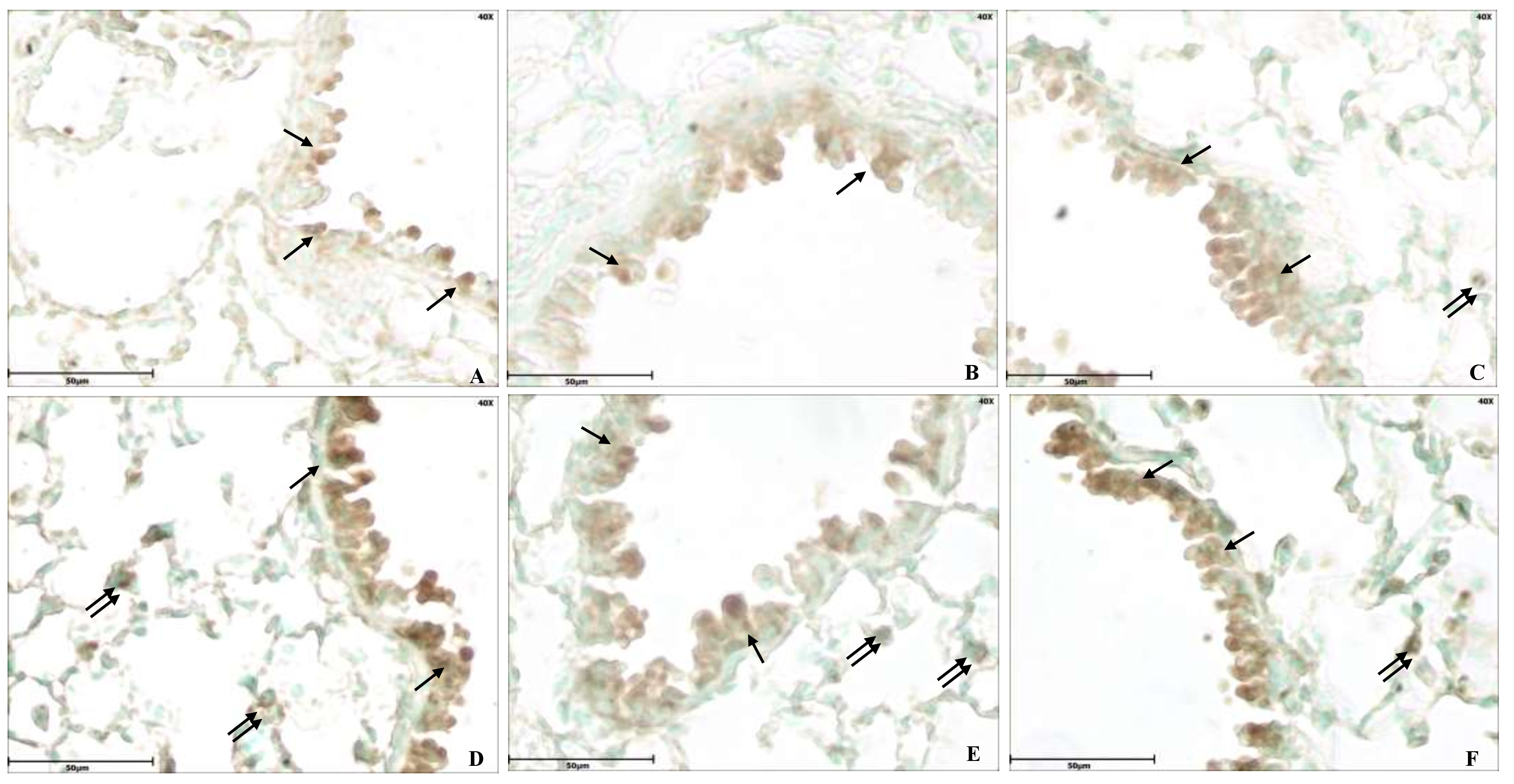

Supplement: Supplementary file 1 — Additional file 1: Figure S1. Immunohistochemistry for expression of p53: Immunopositive reactivity for p53 in alveolar septal cell (single arrow) and epithelium cells (double arrow) in control (A), LPS (B), high dose without LPS (C), low dose without LPS (D), high dose in combination with LPS (E) and low dose in combination with LPS (F) group. Original magnification: 40X. [file 12995_2021_304_MOESM1_ESM.tiff]

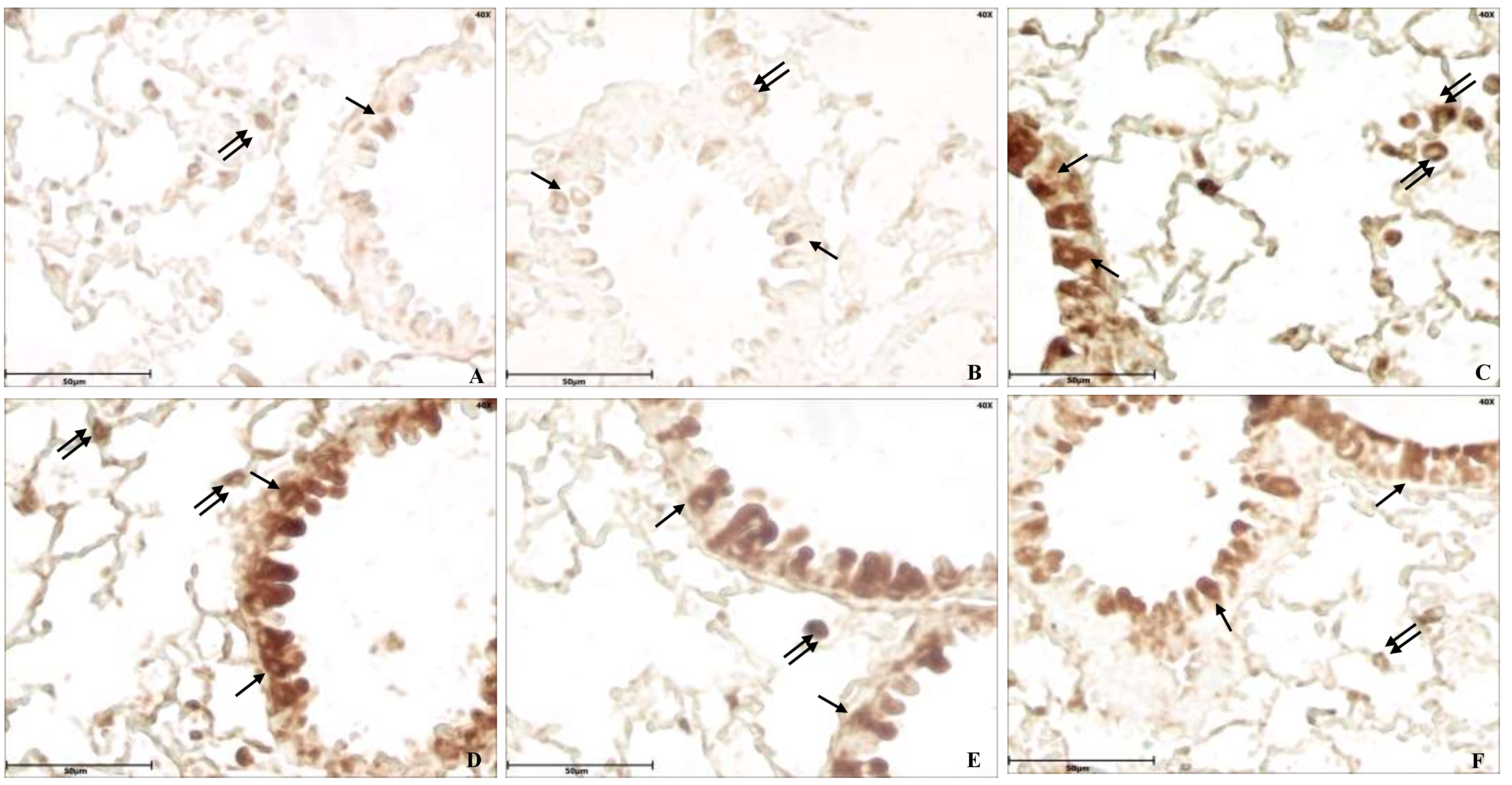

Supplement: Supplementary file 2 — Additional file 2: Figure S2. Immunohistochemistry for expression of Itgb1: Immunopositive reactivity for Itgb1 in alveolar septal cell (single arrow) and epithelium cells (double arrow) in control (A), LPS (B), high dose without LPS (C), low dose without LPS (D), high dose in combination with LPS (E) and low dose in combination with LPS (F) group.. Original magnification: 40X. [file 12995_2021_304_MOESM2_ESM.tiff]

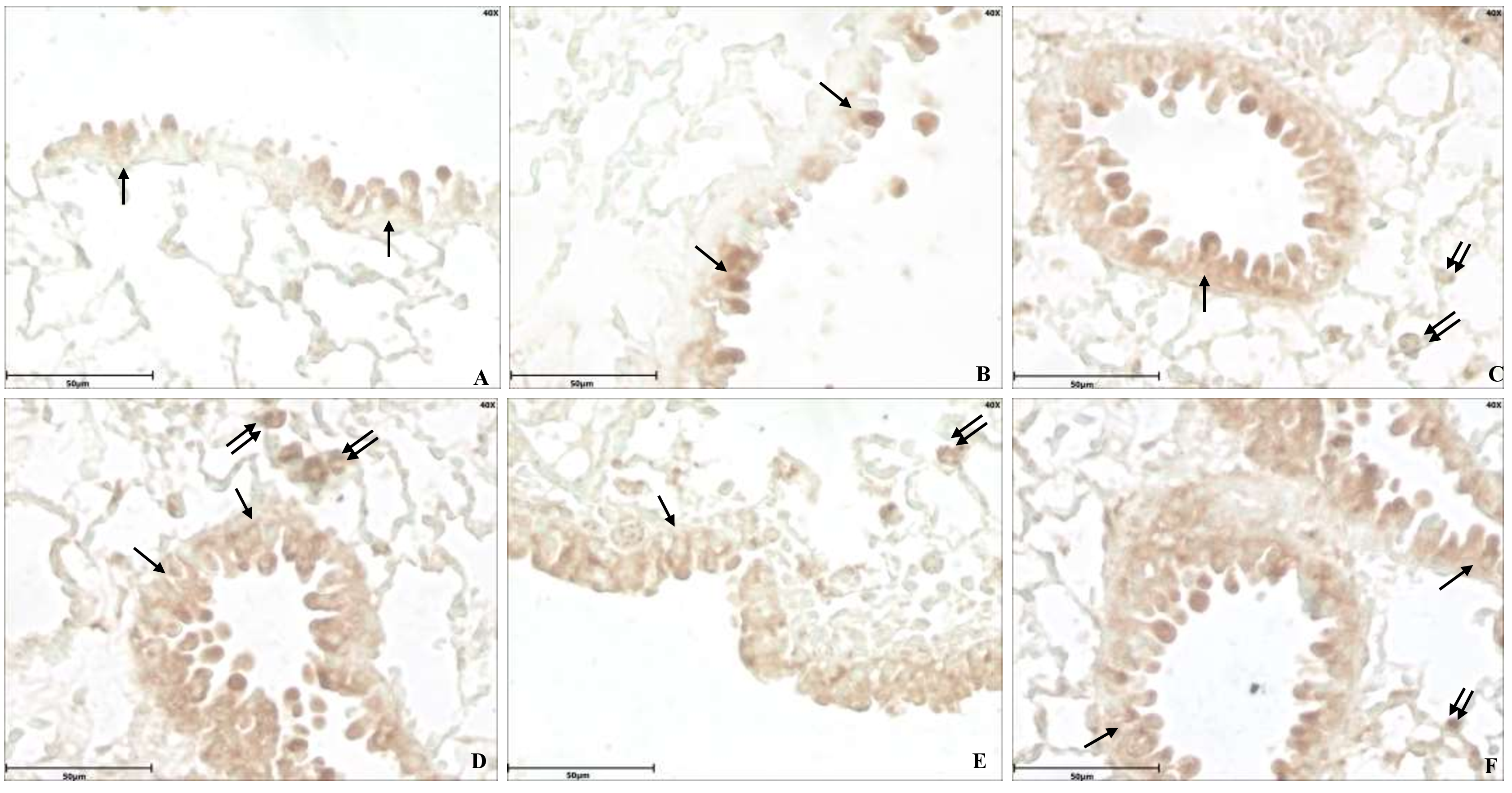

Supplement: Supplementary file 3 — Additional file 3: Figure S3. Immunohistochemistry for expression of Cdk6: Immunopositive reactivity for Cdk6 in alveolar septal cell (single arrow) and epithelium cells (double arrow) in control (A), LPS (B), high dose without LPS (C), low dose without LPS (D), high dose in combination with LPS (E) and low dose in combination with LPS (F) group. Original magnification: 40X. [file 12995_2021_304_MOESM3_ESM.tiff]

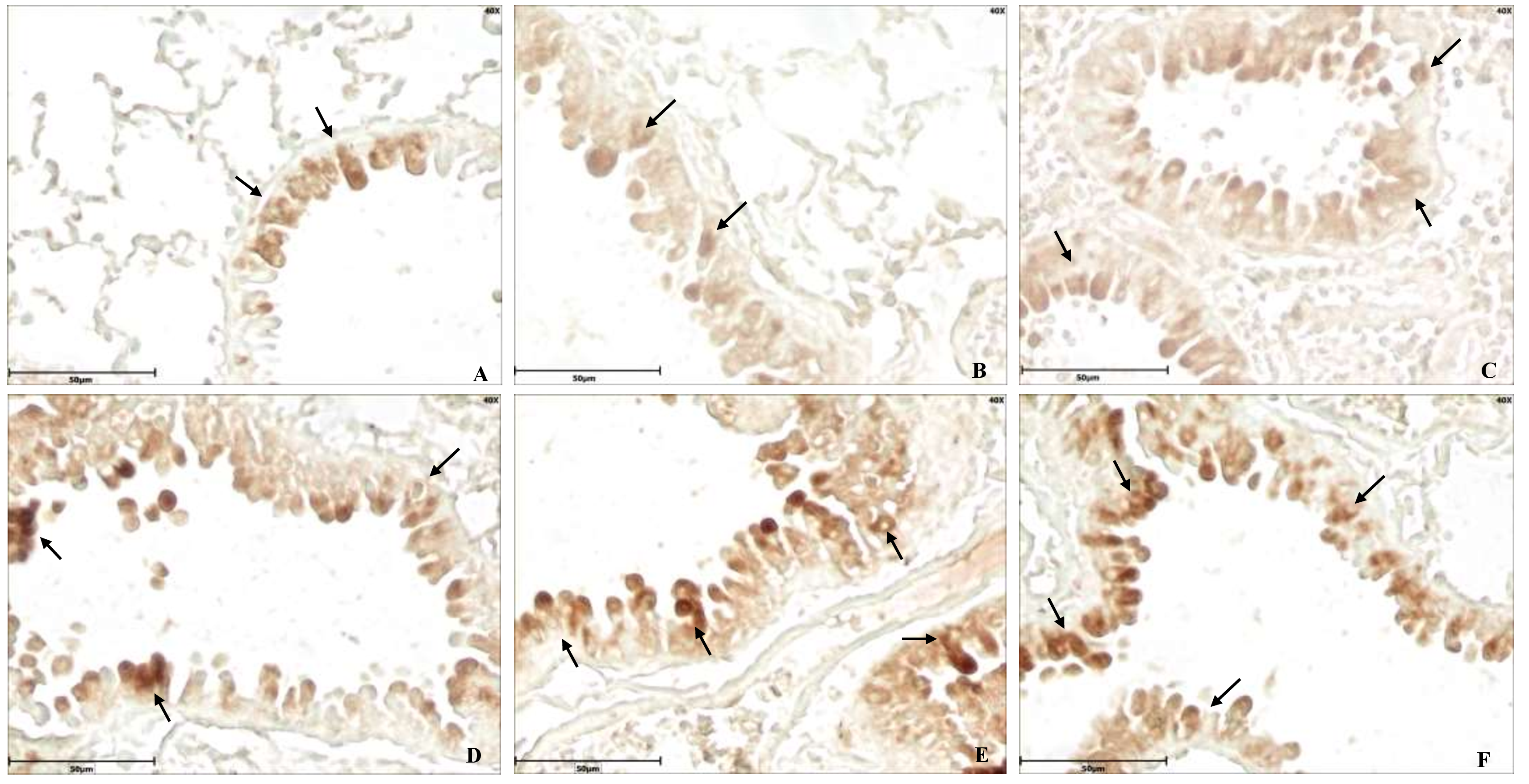

Supplement: Supplementary file 4 — Additional file 4: Figure S4. Immunohistochemistry for expression of Nfkb1: Immunopositive reactivity for Nfkb1 in alveolar septal cell (single arrow) and epithelium cells (double arrow) in control (A), LPS (B), high dose without LPS (C), low dose without LPS (D), high dose in combination with LPS (E) and low dose in combination with LPS (F) group. Original magnification: 40X. [file 12995_2021_304_MOESM4_ESM.tiff]

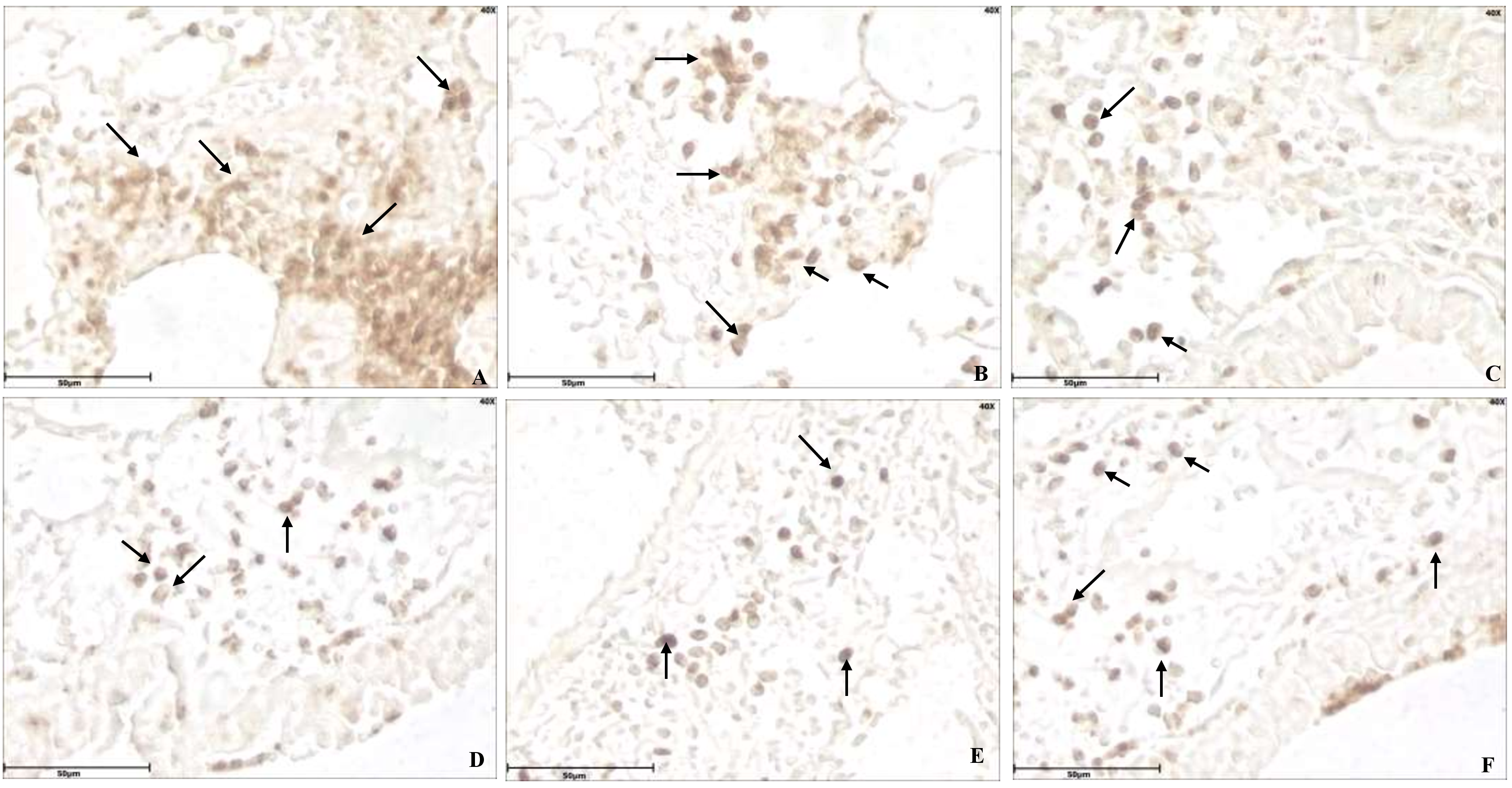

Supplement: Supplementary file 5 — Additional file 5: Figure S5. Immunohistochemistry for expression of Apaf1: Immunopositive reactivity for Apaf1 in alveolar septal cell (single arrow) and epithelium cells (double arrow) in control (A), LPS (B), high dose without LPS (C), low dose without LPS (D), high dose in combination with LPS (E) and low dose in combination with LPS (F) group. Original magnification: 40X. [file 12995_2021_304_MOESM5_ESM.tiff]
