# Supplementary material for: Exposures to 2,4-Dichlorophenoxyacetic acid with or without endotoxin upregulate small cell lung cancer pathway
Source: J Occup Med Toxicol. 2021 Apr 17;16:14. doi: 10.1186/s12995-021-00304-4 (PMC8052721; doi:10.1186/s12995-021-00304-4)
Supplement: Supplementary file 6 — Additional file 6: Table S1. [file 12995_2021_304_MOESM6_ESM.docx]

**Supplementary Table 1: Total Histology Score (THS) for HE-stained lung tissue sections in various groups**

| **Experimental Groups** | **Mean THS** |
| --- | --- |
| Control | 0.61±0.08^a^ |
| LPS | 1.97±0.14^b^ |
| High dose of 2,4-D | 2.16±0.12^b^ |
| Low dose of 2,4-D | 2.0±0.11^b^ |
| High dose of 2,4-D+LPS | 2.36±0.15^b^ |
| Low dose of 2,4-D+LPS | 2.27±0.12^b^ |

Total histology score is expressed as Mean±SE

6 animals from each group were used

^a,b^no common superscript between two levels of an effect indicates significant difference (p<0.05).
