# Supplementary material for: Exposures to 2,4-Dichlorophenoxyacetic acid with or without endotoxin upregulate small cell lung cancer pathway
Source: J Occup Med Toxicol. 2021 Apr 17;16:14. doi: 10.1186/s12995-021-00304-4 (PMC8052721; doi:10.1186/s12995-021-00304-4)
Supplement: Supplementary file 7 — Additional file 7: Table S2. [file 12995_2021_304_MOESM7_ESM.docx]

**Supplementary Table 2: Top five biological processes with enrichment score**

| **S.No.** | **Biological processes** | **Enrichment score** | **P value** |
| --- | --- | --- | --- |
| **1** | Response to oxygen-containing compound (GO:1901700) | 1.3 | 3.59E-02 |
| **2** | Tissue development (GO:0009888) | 1.29 | 3.85E-03 |
| **3** | Regulation of protein modification process (GO:0031399) | 1.29 | 1.52E-03 |
| **4** | Regulation of cell population proliferation (GO:0042127) | 1.26 | 3.37E-02 |
| **5** | Nucleic acid metabolic process (GO:0090304) | 1.25 | 2.31E-02 |
